# Orchestrating agentic systems at ALS beamline 5.3.1 with *Osprey* and *Bluesky*

**DOI:** 10.1107/S1600577526007836

**Published:** 2026-09-01

**Authors:** Ka Hung Chan, Yang Ha, Antoine Islegen-Wojdyla, Seij De Leon, Manuel Garces, Dylan McReynolds, Xiaoya Chong, Raja Vyshnavi Sriramoju, Johannes Mahl, Damian Guenzing, Wiebke Koepp, Alexander Hexemer, Thorsten Hellert, Tanny Chavez

**Affiliations:** a Advanced Light Source Lawrence Berkeley National Laboratory Berkeley CA94720 USA; b Berkeley Center for Structural Biology, Molecular Biophysics and Integrated Bioimaging Lawrence Berkeley National Laboratory Berkeley CA94720 USA; c California Polytechnic State University San Luis Obispo CA93407 USA; d Accelerator Technology and Applied Physics Division Lawrence Berkeley National Laboratory Berkeley CA94720 USA; POSTECH, Republic of Korea

**Keywords:** synchrotron experiment, beamline automation, agentic AI, intent-driven control, workflow portability

## Abstract

Modern synchrotron beamlines support increasingly complex experiments, but their operation remains strongly dependent on facility-specific control interfaces and workflows. This reliance creates a significant barrier to entry for new users and limits the portability of experimental procedures across beamlines. Here, we present a minimal, agent-agnostic orchestration architecture for AI-assisted beamline operation, deployed at Advanced Light Source beamline 5.3.1. The approach introduces a capability-based abstraction layer that exposes beamline functionality as a set of predefined operations. Within this framework, AI agents translate natural-language user requests into structured experimental plans composed of these capabilities. All plans are executed through the *Bluesky Queue Server*, ensuring deterministic operation within existing control and safety constraints without modification of the underlying beamline control system. The framework is defined by three key elements: (i) a portable capability layer that decouples user intent from beamline-specific implementation, (ii) a hybrid interaction framework combining AI-assisted workflow composition with graphical user interface (GUI)-based monitoring and control, and (iii) constrained execution that restricts all actions to predefined capabilities and requires user approval prior to execution. We demonstrate the architecture using grazing-incidence scattering (GISAXS), multi-edge X-ray absorption spectroscopy (XANES), and cross-beamline deployment scenarios. In each case, the system generates physically meaningful experimental plan, adapts to local hardware constraints, and preserves experimental intent across different instruments. These results demonstrate a practical and scalable approach for integrating AI into synchrotron experimentation, enabling intent-driven, portable, and safe beamline operation.

## Abstract 2

A minimal, agent-agnostic AI-assisted orchestration framework for beamline operation that leverages *Osprey*, a production-ready framework for deploying agentic AI in large-scale, safety-critical control-system environments, deployed at Advanced Light Source beamline 5.3.1, is introduced.

## 1. Introduction

Synchrotron radiation enables advanced structural, electronic, and chemical characterization across a wide range of scientific fields, including materials science, condensed matter physics, chemistry, and structural biology (Chantler *et al.*, 2024 ▸; Zhang *et al.*, 2022 ▸; Oberthür *et al.*, 2025 ▸). Modern synchrotron facilities increasingly support complex experimental methods, such as *in situ* and *operando* measurements, automated scanning workflows, and high-throughput data acquisition. While these capabilities significantly expand experimental possibilities, they also introduce substantial complexity in beamline operation and experimental workflow design.

One of the main challenges in beamline operation is the usability of control systems, particularly for new or infrequent users. Learning to operate beamline-specific software often requires significant effort, and users who do not regularly use these systems may find it difficult to maintain familiarity over time. Graphical user interfaces (GUIs) help lower this barrier by providing direct access to common experimental operations, reducing the need for detailed knowledge of underlying control systems. However, GUIs are typically limited to predefined routines and may not support more complex or non-standard experiments. In contrast, scripting environments such as *Bluesky* (Allan *et al.*, 2019 ▸) offer greater flexibility for automation and advanced workflows, but require programming expertise and familiarity with beamline-specific implementations. Within the *Bluesky* ecosystem, services such as the *Bluesky Queue Server* provide reliable execution of serialized experimental plans, while tools such as *Tiled* support structured access to scientific data (NSLS-II, 2025*a* ▸; Bluesky Project, 2026 ▸). These developments have improved programmability and automation at modern beamlines, but they do not by themselves remove the need for users to translate high-level experimental goals into beamline-specific procedures. Similar challenges have motivated the development of dedicated beamline control systems at other facilities, such as BLISS at the ESRF, which was designed explicitly to provide a more accessible and flexible experiment control environment for a diverse user community (Guijarro *et al.*, 2023 ▸).

A growing body of work has addressed these challenges through the development of autonomous and adaptive experimentation. By embedding machine learning (ML) and optimization algorithms directly into synchrotron workflows, these approaches have proven highly effective for automating beamline alignment (Morris *et al.*, 2024 ▸) and driving closed-loop data acquisition (Beaucage & Martin, 2023 ▸; Wang *et al.*, 2025 ▸). Autonomous laboratory platforms extend these capabilities to enable ML-driven closed-loop workflows for materials synthesis and characterization. Recent cross-facility work demonstrates that autonomous scattering workflows can be seamlessly deployed across different beamlines with minimal modification. This portability is achieved by constructing the workflows from modular components for data access, orchestration, analysis, visualization, and adaptive decision-making (Koepp *et al.*, 2026 ▸). Complementary work at NSLS-II has leveraged the hardware-agnostic *Bluesky* infrastructure to seamlessly orchestrate multi-modal, multi-beamline experiments in real time using an ensemble of AI agents for on-the-fly data reduction and Bayesian-driven acquisition (Corrao *et al.*, 2025 ▸). These efforts demonstrate the potential of modular autonomous experimentation to improve efficiency and reproducibility, especially for well defined experimental classes such as scattering.

In parallel, recent advances in AI, particularly agent-based systems and large language models (LLMs), have opened a complementary direction for beamline interaction. By enabling natural-language interaction, AI agents can translate high-level user intent into structured experimental procedures, reducing the need for direct interaction with complex control interfaces. Early demonstrations in scientific workflows suggest that such systems can lower the barrier to complex experiment design while preserving flexibility (Mathur *et al.*, 2025 ▸; Prince *et al.*, 2024 ▸). More recent work has shown AI agents operating directly on synchrotron instrumentation, including diffractometers, X-ray nanoprobes, and particle accelerators (Kaiser *et al.*, 2025 ▸; Chen *et al.*, 2025 ▸; Vriza *et al.*, 2026 ▸). By allowing users to express experimental goals in natural language, agentic systems decouple what a user wants to achieve from how a specific beamline implements that request, a separation that neither GUIs nor scripting environments provide on their own.

While efforts such as *Osprey* have begun to address safe agentic operation in accelerator control rooms (Hellert *et al.*, 2026*b* ▸), translating this potential into general beamline operation requires capabilities extending beyond natural-language plan generation. These include a representation of beamline capabilities that is portable across instruments, an execution pathway that remains compatible with existing control infrastructure, mechanisms for preventing invalid or physically inconsistent actions, and user-facing tools for approval, monitoring, and intervention during execution. Traditional autonomous experiment frameworks avoid these problems by operating strictly within rigid scientific workflows, optimization loops, or measurement modalities. Conversely, to safely leverage the flexibility of agentic interfaces, these models must be carefully constrained before they can be trusted to interact with real instruments. While existing tools address some of these requirements in controlled settings, no prior system has integrated them into a deployable framework for general beamline operation. This assessment is echoed by a recent community workshop on agentic AI at scientific user facilities, which identified portability, safe execution, human oversight, and observability as the outstanding open challenges for practical deployment (Hellert *et al.*, 2026*a* ▸).

To address these challenges, we introduce a minimal, agent-agnostic AI-assisted orchestration framework for beamline operation that leverages *Osprey*, a production-ready framework for deploying agentic AI in large-scale, safety-critical control-system environments (Hellert *et al.*, 2026*b* ▸). Demonstrated at the Advanced Light Source (ALS) beamline 5.3.1, our system enables users to specify experiments in natural language and translates those requests into structured workflows expressed using predefined beamline capabilities. These workflows are executed through the *Bluesky Queue Server* (NSLS-II, 2025*a* ▸), preserving deterministic execution and existing control-system constraints without requiring modification of the underlying beamline control system. This work makes three contributions. First, we introduce a capability-based abstraction that decouples experimental intent from beamline-specific implementation, enabling portable and consistent interaction across instruments. Second, we employ a hybrid interaction paradigm through the *Finch* GUI (NSLS-II, 2025*b* ▸), where AI agents compose experimental workflows while the GUI provides real-time monitoring and user control. Third, we establish a constrained execution environment for the AI agent to ensure safe and reliable beamline operation by restricting actions to predefined capabilities and requiring user approval prior to execution.

We demonstrate the proposed architecture through some representative case studies, including grazing-incidence small-angle scattering (GISAXS), multi-edge X-ray near-edge absorption spectroscopy (XANES), and cross-beamline deployment scenarios. These examples illustrate how the system supports intent-driven, consistent, and safe experimental workflows across different beamline environments, providing a practical framework for integrating AI into synchrotron experimentation. The remainder of this paper is organized as follows: Section 2 introduces the control environment at beamline 5.3.1, Section 3 describes the system architecture and implementation, Section 4 presents the case studies, and Section 5 presents the conclusions and directions for future work.

## 2. Beamline 5.3.1 control environment

Beamline 5.3.1 at the ALS is a tender X-ray beamline operating over an energy range of 2.45–12 keV using a bending-magnet source. It supports a range of experimental modalities, including X-ray scattering, spectroscopy, and instrumentation development (Voronov *et al.*, 2025 ▸; Allan *et al.*, 2019 ▸). To support automated and high-throughput experiments, the beamline has adopted a control system based on *Experimental Physics and Industrial Control System* (*EPICS*) (EPICS Collaboration, 2026 ▸) for hardware control and the *Bluesky* framework for experiment orchestration (Allan *et al.*, 2019 ▸). This capability has been demonstrated at the beamline through automated beamline alignment based on Bayesian optimization (Morris *et al.*, 2024 ▸).

**Figure 1:**
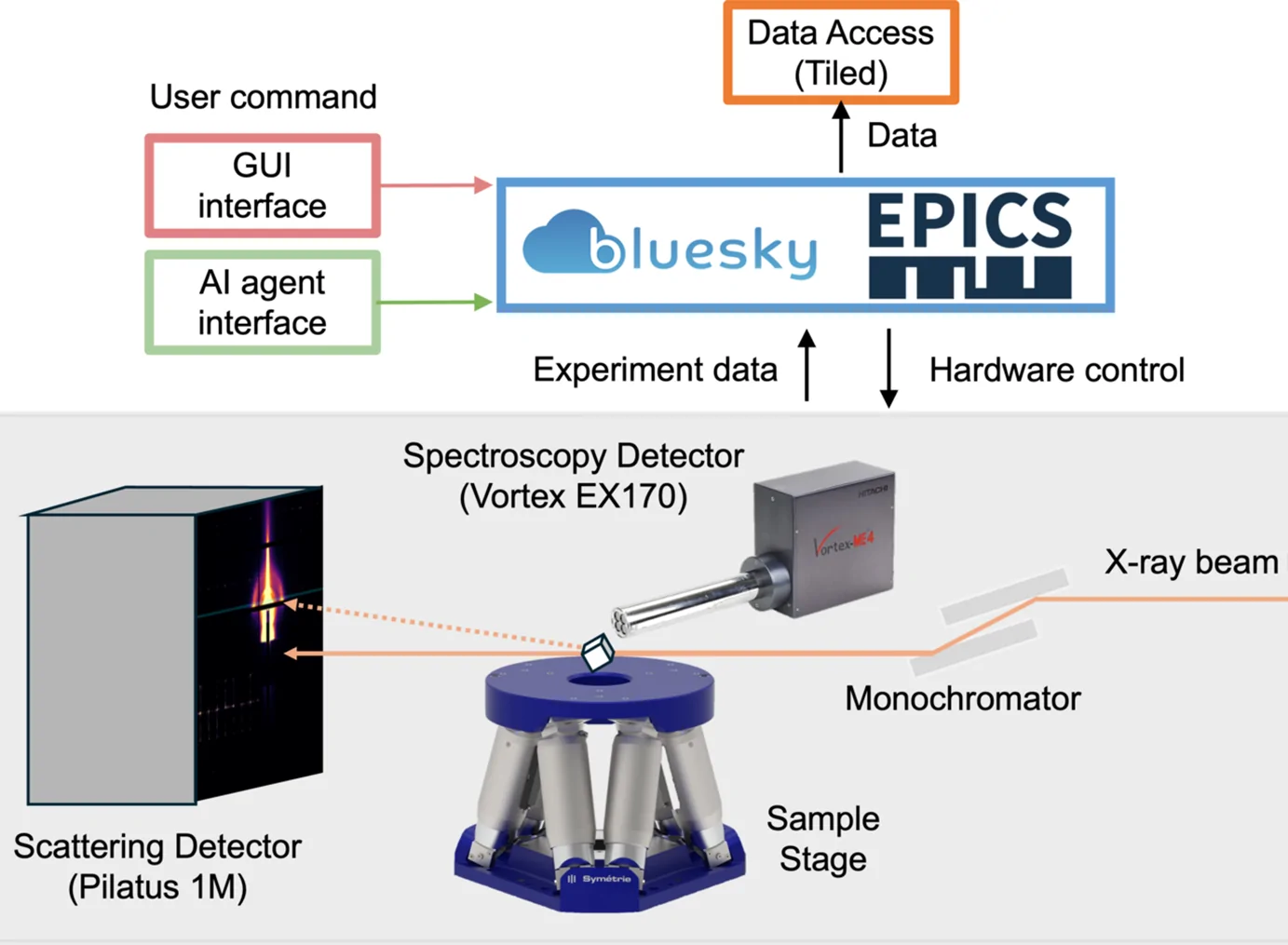
Overview of ALS beamline 5.3.1. (Top) User interfaces (GUI and AI agent) interact with the beamline through the *Bluesky–EPICS* control system. Data are managed through *Tiled*. (Bottom) Physical layer showing the monochromator, hexapod sample stage, and detectors for spectroscopy and scattering. All measurements follow the same control pathway.

As illustrated in Fig. 1 ▸, this control system comprises *EPICS*, the *Bluesky* framework, the *Bluesky Queue Server* (NSLS-II, 2025*a* ▸), and the *Tiled* data server (Bluesky Project, 2026 ▸). *EPICS* provides direct communication with beamline hardware, including motors and detectors. The *Bluesky* framework defines experiments as structured plans that coordinate device operations and data acquisition. These plans can be executed interactively or through the *Bluesky Queue Server* (Allan *et al.*, 2019 ▸; NSLS-II, 2025*a* ▸), which manages execution by serializing requests and checking them against beamline constraints to ensure safe and consistent operation. Experimental data are stored and accessed through the *Tiled* server (Bluesky Project, 2026 ▸), which provides a unified interface for data retrieval and analysis.

To support routine beamline operation and reduce the complexity of direct interaction with control systems, a GUI, *Finch*, is under active development at the ALS (NSLS-II, 2025*b* ▸; Leon *et al.*, 2025 ▸). *Finch* is a modular *React*-based component library designed for *Bluesky* beamlines (Leon *et al.*, 2025 ▸). Following atomic design principles, it provides reusable, nestable components that can be composed into beamline-specific interfaces, while a shared ALS style guide ensures a consistent user experience across beamlines. Key components include a *Tiled* data viewer for real-time access to experimental data stored on *Tiled* servers and a *Queue Server* interface for submitting and monitoring experimental plans (Bluesky Project, 2026 ▸; NSLS-II, 2025*a* ▸). *Finch* provides users with direct access to common experimental procedures, real-time visualization of detector data, and control of beamline components. It offers a stable and intuitive environment for standard measurements and serves as the primary interface for monitoring experiment status during execution.

In practice, users operate beamline 5.3.1 through a combination of graphical and scripting interfaces. Routine tasks, such as instrument adjustment and alignment, are typically performed through the *Finch* GUI, which provides convenient access to control and immediate feedback. Standard experimental procedures are executed through the *Bluesky Queue Server* using predefined plans submitted via the *Finch* GUI, while more complex or customized experiments are often carried out through scripting environments such as Python notebooks. This hybrid interaction framework allows users to balance ease of use with experimental flexibility while maintaining a consistent execution pathway.

However, while scripting interfaces provide flexibility, they require programming expertise and introduce additional effort in specifying and managing experimental workflows. This can present a barrier for users with limited programming experience and increase cognitive load even for experienced users. These challenges motivate the development of higher-level interaction models in which users specify experimental intent, while an AI agent assists in translating this intent into executable operations.

## 3. Architecture and implementation

**Figure 2:**
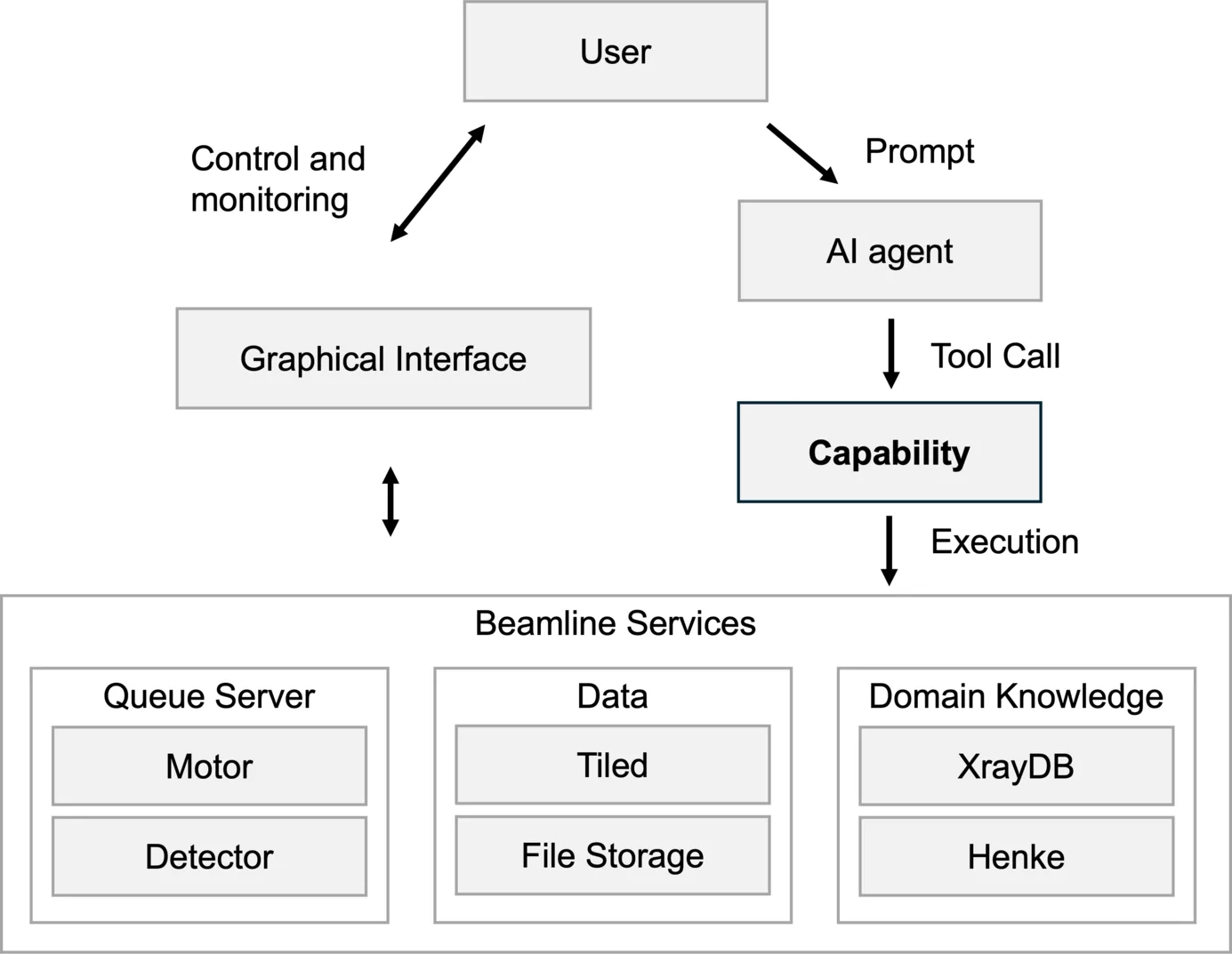
Architecture of the AI-assisted beamline orchestration framework. User interaction occurs through both a graphical interface and an AI agent. The AI composes workflows from high-level intent using predefined capabilities, while the GUI provides direct control and real-time monitoring. The capability layer abstracts beamline operations and interfaces with shared beamline services, including hardware control, data access, and domain knowledge resources. All actions are executed within the same validated control environment.

An overview of the proposed orchestration architecture is shown in Fig. 2 ▸. The system introduces an intent-driven interaction model in which high-level user requests are translated into structured capability calls.

We implement this framework at beamline 5.3.1 using the *Osprey* architecture, and the *Finch* GUI. *Osprey* is a production-oriented AI-agent framework designed for safety-critical scientific control environments, including accelerators, beamlines, and large-scale experimental facilities (Hellert *et al.*, 2026*b* ▸). It is built around a plan-first orchestrator that generates complete execution plans for human review before any hardware action is taken, a coordination layer that manages data flow and type consistency, and a dynamic tool selector that restricts the agent to only the capabilities required for a given task (Hellert *et al.*, 2026*b* ▸). Additionally, the system incorporates established domain knowledge by giving the agent access to the *xraydb* Python library and the Henke database (Henke *et al.*, 1993 ▸; Newville *et al.*, 2025 ▸). In this work, *Osprey* operates strictly as a workflow coordination layer that interprets user intent and composes experimental workflows. Rather than interfacing with *EPICS* directly, all hardware actions are dispatched through the *Bluesky Queue Server*, ensuring all actions follow a consistent, controlled execution path.

### 3.1. Capability-based abstraction and portability

The core of the framework is a capability-based abstraction layer that exposes beamline functionality as a set of pre­defined operations. As shown in Table 1 ▸, each capability represents a common experimental or computational action, such as motor motion, data acquisition, data access, or analysis, and is defined through a structured interface. Rather than issuing device-specific commands, the AI agent maps natural-language user requests onto sequences of these capabilities, which are invoked through structured tool calls with explicit, machine-readable parameters.

This design decouples high-level experimental intent from beamline-specific implementation. Each capability consists of two components: a capability description and a backend implementation. The description defines the operation, input parameters, and valid ranges or constraints, allowing the AI to determine how and when to use each capability when composing workflows. The backend implementation maps the capability to beamline-specific operations and may interact with hardware control systems, data services, or analysis routines, depending on the specific beamline environment.

A key advantage of this design is portability across beamlines. Because capability descriptions remain consistent while implementations are adapted locally, the same high-level user request can be applied across instruments with different hardware configurations and control systems. For example, a user may request: ‘move the sample up by 1 mm’. While the intent is identical, different beamlines may use different motor names, coordinate systems, or units. The capability translates this request into the appropriate device commands and parameters, ensuring consistent behavior without requiring the user to understand the underlying hardware. As a result, the same user prompt produces equivalent experimental actions across beamlines, with hardware and control differences handled transparently by the system.

More broadly, this abstraction enables a unified interface for synchrotron experimentation. A single user prompt can be consistently interpreted and adapted across different beamlines, allowing AI agents to operate at the level of experimental intent rather than hardware-specific commands. This provides a pathway toward standardized, intent-driven control of experiments across facilities while remaining compatible with existing control systems and safety constraints.

### 3.2. Hybrid interaction framework

Another contribution of this work is a hybrid interaction framework that combines AI-assisted workflow composition with GUI-based monitoring and control through *Finch*. In this approach, AI is used to construct complex, multi-step experimental workflows from high-level user intent, while direct manipulation of beamline components and routine operations are handled through the GUI

**Figure 3:**
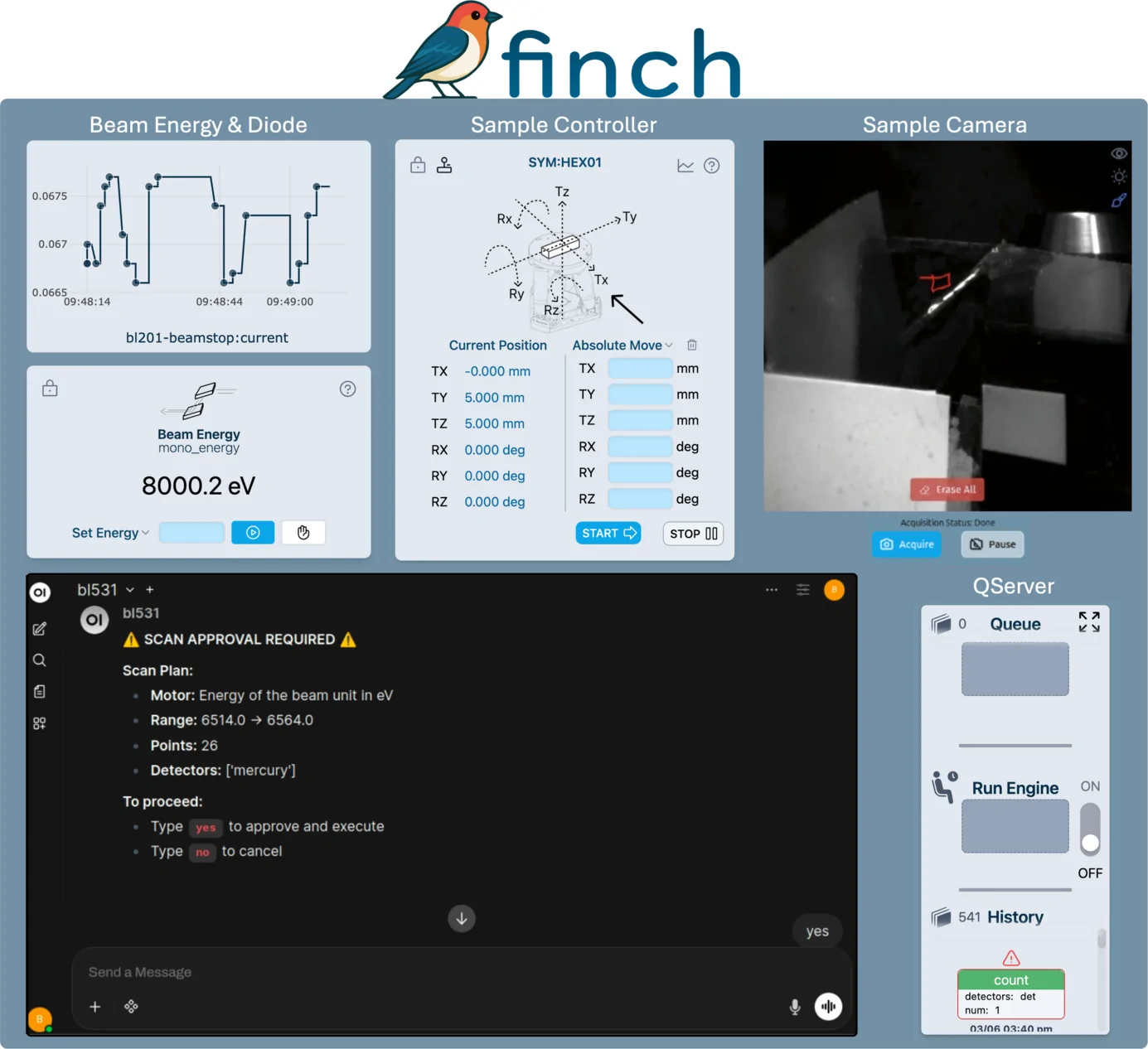
Hybrid beamline interface (*Finch*) combining graphical monitoring and AI-assisted orchestration. The GUI provides real-time control and visualization (beam status, detector images, sample positioning, and queue status), while the integrated AI interface enables natural-language workflow composition and execution. Both interaction modes operate through the same validated control pathway.

As illustrated in Fig. 3 ▸, the *Finch* GUI provides real-time visualization of detector data and direct control of beamline components, while also presenting AI-generated plans for user review. Users can monitor execution, adjust parameters, and pause or terminate experiments at any time, ensuring continuous oversight and control during operation.

This design aligns with how beamline experiments are typically carried out in practice, where users rely on graphical interfaces for direct control and real-time feedback to validate measurements and intervene when needed. Simple actions, such as adjusting motor positions or inspecting detector images, are more efficiently performed through graphical interfaces, whereas AI assistance becomes valuable for coordinating multi-step procedures, selecting parameters, and adapting workflows to experimental conditions. By combining these interaction modes, the system reduces interaction overhead, simplifying experimental setup while maintaining flexibility and user control.

### 3.3. Safety and constrained execution

Safe and rigorous operation is enforced through multiple layers of constraints spanning hardware, control software, AI reasoning, and user oversight. At the hardware level, beamline components are governed by controller limits and hard stops that prevent operations outside safe physical ranges. These constraints provide the fundamental safety boundary for all experiments. At the control system level, actions are executed through *EPICS* and the *Bluesky Queue Server*, where beamline constraints (*e.g.* motor limits, energy ranges, device availability) are enforced. This ensures that all hardware operations remain within allowable operating conditions.

Complementing these safeguards, the AI agent also enforces validation at the reasoning stage. Requests that are incomplete, ambiguous, or physically inconsistent are not translated into executable actions. Instead, the agent requests clarification or proposes physically meaningful alternatives before any plan is executed. This ensures that invalid or unsafe operations are filtered at the intent level, prior to interaction with the control system.

To ensure scientific rigor during this reasoning process and prevent the agent from hallucinating physical parameters, the abstraction layer incorporates domain-knowledge capabilities. Rather than relying on the language model’s internal knowledge to determine values such as absorption edges or atomic scattering factors, the system provides tools that query the *xraydb* Python library and the Henke database (Henke *et al.*, 1993 ▸; Newville *et al.*, 2025 ▸), well established references for X-ray interactions with matter. This guarantees that the generated experimental plans are parameterized using accurate values, ensuring rigorous scientific execution.

Finally, at the user interaction level, execution can require explicit user approval before submission. The GUI provides full visibility into proposed plans, parameter values, and execution status. This allows users to inspect, modify, or reject actions before they are carried out. Users can also interrupt or terminate execution at any stage, providing an ultimate layer of human control.

## 4. Case studies: AI-assisted beamline operation

Beamline experiments require users to define experimental goals, construct multi-step workflows, and adapt them to beamline-specific control systems. This process demands both domain expertise and familiarity with local instrumentation, and is often time-consuming and error-prone. In this section, we demonstrate how the *Osprey* framework simplifies this process by allowing users to specify experimental intent directly. The system translates these requests into executable workflows and adapts them to the local beamline environment, reducing the need for manual configuration and scripting.

To make the framework practical for beamline deployment without dedicated local AI hardware, the agent accesses LLM services through configurable external providers while beamline orchestration remains within the local control environment. In the implementation evaluated here, the local agent services ran on a Linux workstation with eight CPU cores and 62 GiB of memory, and model access was provided through the CBorg AI platform. CBorg is a multi-model AI platform developed by the Information Technology Division at Lawrence Berkeley National Laboratory that provides secure access to commercial and Lab-hosted AI models through a common interface (Lawrence Berkeley National Laboratory, IT Division, 2026 ▸). Anthropic Claude Sonnet 4.6 was used for workflow orchestration, whereas OpenAI GPT-4o supported the remaining agent components. Under this configuration, a typical alignment, XANES, data-retrieval cycle consumed approximately 75000 input tokens and 3600 output tokens. For a representative 60-sample beam time, the projected consumption was approximately 4.5–5.9 million input tokens and 0.21–0.27 million output tokens, depending on the session-management strategy. Detailed token-accounting procedures, variability analyses, and comparisons among session-management strategies are provided in the supporting information.

This same deployment configuration was used throughout the case studies to provide a consistent basis for comparison. The experimental GISAXS and XANES studies at ALS beamline 5.3.1 evaluated workflow composition and execution under actual beamline conditions, whereas the simulated cross-beamline study evaluated portability and adaptation to different control environments. Together, these examples show how the framework can be deployed in operational beamline environments to compose, execute, and adapt complex workflows through natural-language interaction while preserving the existing beamline control pathway.

### 4.1. Intelligent GISAXS alignment and acquisition

This example illustrates how the orchestration architecture translates high-level experimental intent into a structured workflow using predefined capabilities. GISAXS is widely used to probe nanoscale morphology in thin films, where accurate sample alignment and appropriate selection of the incidence angle are critical for obtaining meaningful scattering signals. In conventional operation, these parameters are determined through manual estimation and iterative adjustment, requiring both domain expertise and familiarity with beamline-specific procedures.

In the AI-assisted workflow, the user specifies the experimental objective at a high level, for example performing a GISAXS measurement on a polymer thin film on silicon. The AI agent interprets this request and constructs a multi-step workflow composed of predefined capabilities. In this case, the workflow consists of an automated alignment step using the 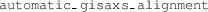 capability. After alignment, the agent invokes the scan capability to perform an angular scan of the grazing-incidence motor from 0.25° to 0.55° with 31 steps while simultaneously acquiring detector images at each position.

The selection of acquisition parameters is guided by domain knowledge embedded within the system. For instance, at a photon energy of 3.5 keV, the agent proposes an initial incidence angle of 0.37° and an angular scan over the range 0.25° to 0.55°, consistent with standard experimental strategies for balancing surface sensitivity and signal intensity. All proposed parameters are presented to the user for confirmation and validated against beamline constraint before execution.

During execution, the graphical interface provides real-time visualization of detector images as the incidence angle is varied. The optimal incidence angle is determined by observing the evolution and contrast of scattering features across the angular scan, allowing the user to identify conditions that maximize surface-sensitive signal. In this demonstration, the AI agent composes the workflow while the GUI remains essential for monitoring, interpretation, and user validation.

**Figure 4:**
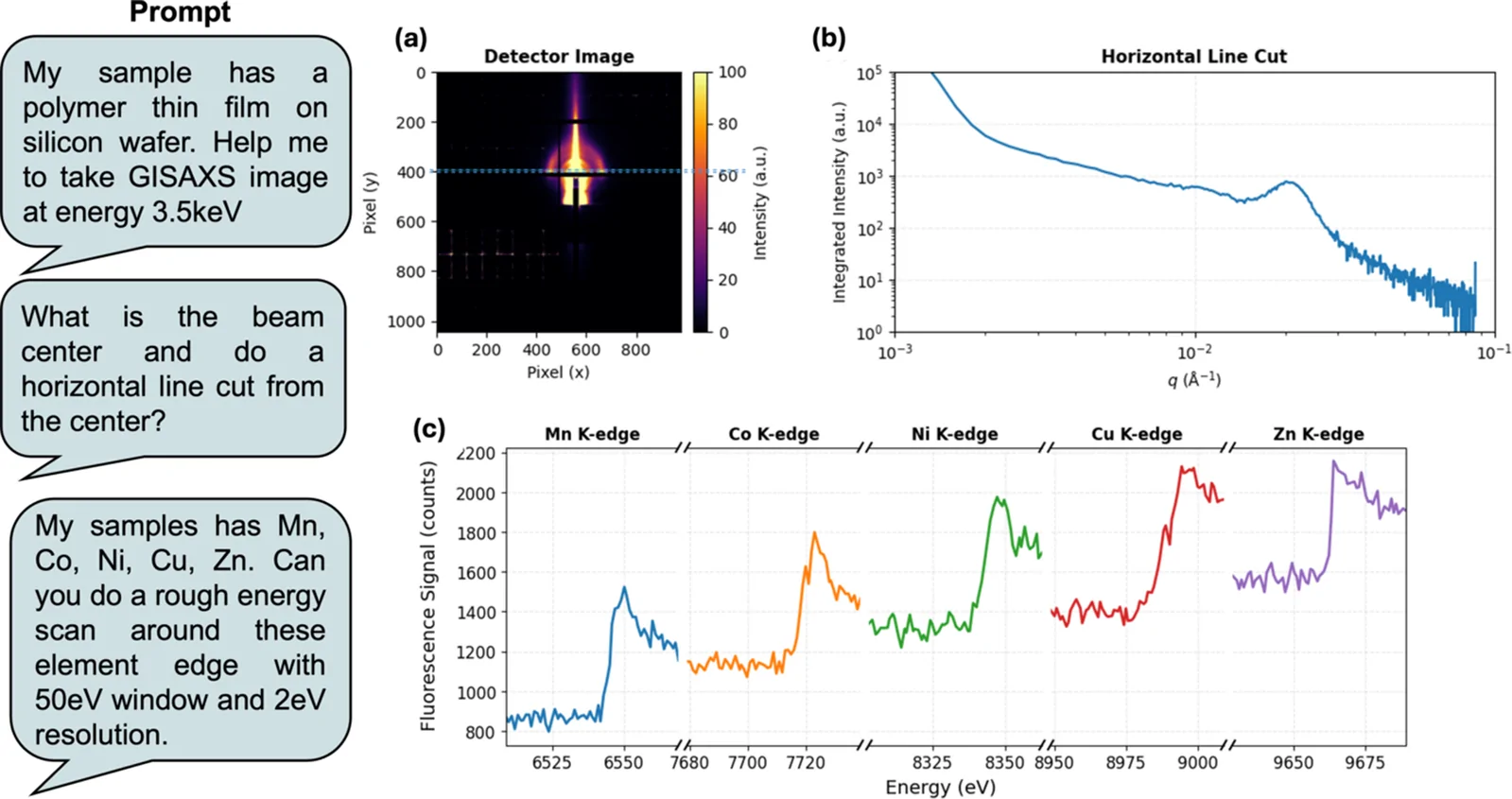
AI-assisted beamline operation at ALS beamline 5.3.1 combining multi-edge fluorescence spectroscopy and tender GISAXS. (*a*) Representative GISAXS detector image acquired during GISAXS scan. (*b*) Corresponding horizontal line cut showing integrated intensity versus momentum transfer *q*. (*c*) Sequential fluorescence scans across Mn, Co, Ni, Cu, and Zn *K*-edges, demonstrating automated edge selection and parameter configuration. All measurements were executed through validated plan templates via the *Bluesky Queue Server*, illustrating coordinated multi-step alignment and spectroscopy within a deterministic control framework.

From the acquired dataset, an optimal incidence angle of approximately 0.45° is identified, which has the maximum signal-to-noise ratio. Following acquisition, the user requested to find the beam center and do a horizontal line cut. The system retrieves the dataset from the *Tiled* server, determines the beam center, and performs the line cut to generate an intensity profile (*I* versus *q*) using a predefined analysis pipeline. These post-processing steps are also implemented as capabilities, demonstrating that the same abstraction framework extends beyond data acquisition to analysis. The results are presented in Figs. 4 ▸(*a*) and 4(*b*).

Compared with conventional workflows, this approach reduces manual parameter tuning and iterative alignment while maintaining transparency and user control. Because all steps are defined through structured capability calls, the workflow can be reproduced and reused across experiments with minimal modification. This example demonstrates how the capability-based abstraction enables the AI agent to construct and execute a complex experimental workflow, while the shared execution pathway and hybrid interaction framework ensure safe, transparent, and user-centered operation.

### 4.2. Multi-edge XANES orchestration

This example demonstrates how the orchestration architecture constructs and executes multi-step spectroscopic workflows through capability composition. XANES measurements are commonly used to probe the electronic structure and chemical state of materials. For samples containing multiple elements, multi-edge measurements are required, which involve repeated manual configuration of scan parameters for each element.

In the AI-assisted workflow, the user specifies the target elements at a high level, for example requesting XANES measurements for a set of transition metals. The AI agent interprets this request and constructs a sequence of capability calls that define the full measurement workflow. This includes retrieving absorption edge energies using the 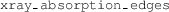 capability and generating parameterized scan operations using the scan capability for each element.

For each element, the system retrieves the corresponding absorption edge energy and defines a scan range ±30 eV around the edge with 61 steps as requested by the user. Similar to the GISAXS example, all proposed parameters are presented to the user for confirmation and validated against beamline constraint before execution

During execution, the graphical interface provides real-time feedback on incident energy and detector response, allowing the user to monitor the evolution of absorption features. This enables users to track experiment progress, interpret results as they are acquired, and adjust experimental parameters when necessary. In this hybrid interaction framework, the AI agent automates the setup of multi-edge XANES measurements, while the GUI allows users to track absorption features in real-time and interpret the data as it is acquired.

The system also handles physically inconsistent inputs at the agent level before execution. For example, if a user requests an Mn XANES scan at an incorrect energy (*e.g.* 25 keV), the AI agent identifies the inconsistency and determines the appropriate Mn *K*-edge energy (approximately 6539 eV). A corrected scan range is then proposed to the user based on domain knowledge, rather than being executed directly. This ensures that requests are physically consistent before a plan is generated and presented to the user for approval.

The resulting spectra exhibit well defined absorption edges at the expected energies, as shown in Fig. 4 ▸(*c*), confirming that the system correctly translates user intent into executable experimental procedures. For comparison, an experienced user independently prepared and executed the same multi-edge XANES experiment using direct *Bluesky* commands. Retrieving the relevant absorption-edge energies, selecting the scan parameters, and preparing the corresponding *Bluesky* plans required approximately 4 min, whereas the AI-assisted workflow completed the same preparation in approximately 1 min. The physical data-acquisition time was unchanged because both approaches executed equivalent validated *Bluesky* plans with the same scan parameters.

This example demonstrates how the orchestration architecture enables automated multi-step spectroscopic measurements, ensuring accurate and reproducible acquisition of element-specific absorption edges at the correct energies. The combined design maintains physically consistent operation while supporting real-time user monitoring and interpretation.

### 4.3. Cross-beamline portability and deployment

This section highlights how the capability-based abstraction decouples experimental intent from beamline-specific implementation, enabling portable workflows across instruments with different hardware configurations. In conventional operation, experimental parameters such as X-ray energy and sample–detector distance must be manually configured based on beamline-specific constraints and sample properties. As a result, translating a scientific objective, such as probing a target length scale, into instrument settings requires detailed knowledge of the local system.

In the AI-assisted workflow, the user instead specifies the experimental objective directly. For example, a request to probe structural features in the range 250–500 Å corresponds to a target momentum transfer range of approximately *q* ≃ 0.013–0.025 Å^−1^. The AI agent interprets this intent and constructs a workflow using predefined capabilities, while the underlying implementation adapts to beamline-specific constraints.

**Figure 5:**
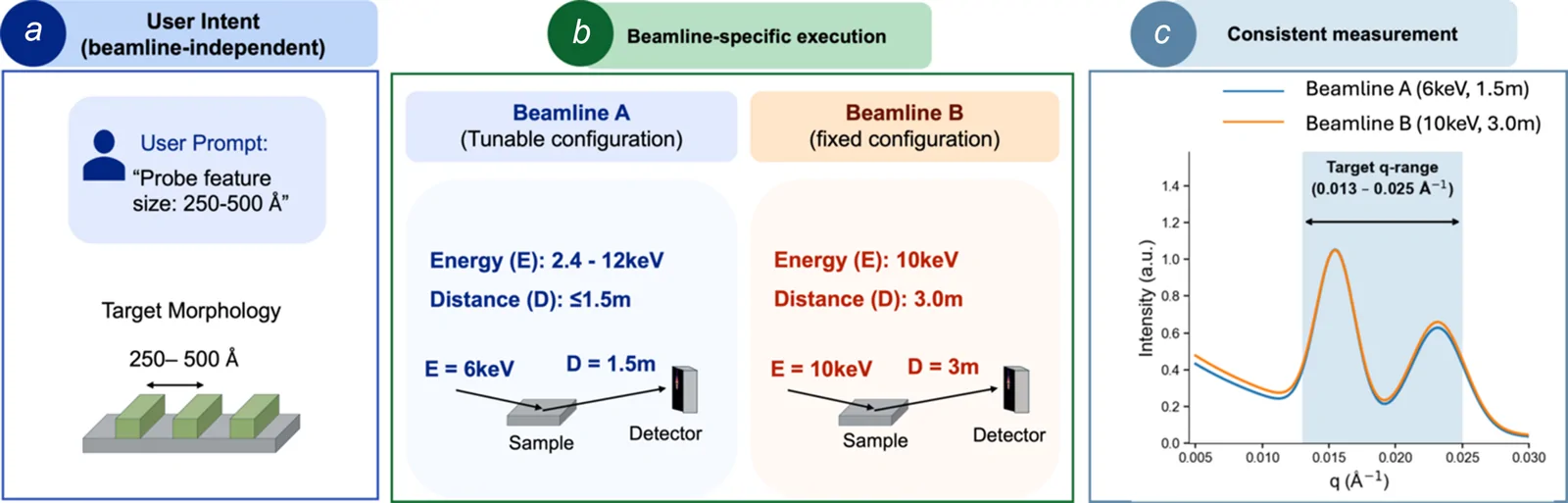
Schematic illustration of cross-beamline portability enabled by intent-based control. (*a*) A beamline-independent user request specifies a target structural length scale. (*b*) The system translates this intent into beamline-specific configurations, adapting parameters such as X-ray energy and sample–detector distance according to local constraints. (*c*) Despite different configurations, the resulting measurements probe the same momentum transfer (*q*) range, yielding consistent structural information.

To illustrate this behavior, we consider two simulated beamline endstations with different configurations, as shown in Fig. 5 ▸. One configuration is based on the operational parameters of ALS beamline 5.3.1, while the second is based on beamline 7.3.3. At beamline A, which supports variable energy (5–12 keV) and adjustable sample–detector distance up to approximately 1.5 m, after calculation and reasoning, the system selects an energy of 6 keV (λ ≃ 2.07 Å) and a distance of 1.5 m to achieve the desired *q*-range. The workflow is then constructed using capability calls for energy selection, detector positioning, alignment, and data acquisition.

At beamline B, the same request is handled differently due to fixed hardware constraints. This beamline operates at a fixed energy of 10 keV (λ ≃ 1.24 Å) and a fixed detector distance of approximately 3.0 m. The system evaluates the requested *q*-range under these constraints and determines that the target structural features are accessible without modification of hardware parameters. The workflow therefore proceeds directly with alignment and data acquisition using the available configuration.

In both cases, the workflow is expressed using the same set of capabilities, and the underlying *Bluesky* plan logic remains unchanged. Portability is achieved by redefining beamline-specific components, such as motors, detectors, and operational parameters. The AI agent uses these configurations to generate appropriate plans for each beamline, which are then validated and executed through the *Queue Server* to ensure safe and consistent operation.

As a result, the same high-level user request produces different execution strategies depending on the beamline configuration. A beamline with variable energy and adjustable sample–detector distance may optimize both parameters to achieve a target *q*-range, whereas a beamline with fixed energy and geometry operates within its predefined configuration and evaluates feasibility accordingly. In both cases, the experimental intent is preserved while execution details adapt to local constraints.

The framework also handles unsupported requests in a beamline-dependent manner. For example, a user may request an energy-dependent measurement near the Cu *K*-edge. On beamline A with tunable energy, the agent generates and executes the appropriate scan. In contrast, on beamline B with fixed energy, the agent identifies that the request cannot be fulfilled and informs the user that the current energy does not match the Cu absorption edge. Rather than generating an invalid plan, the agent requests clarification or suggests alternative measurements.

This example demonstrates that portability is achieved at the capability level, where experimental intent is preserved while execution details adapt to local beamline constraints. By separating experimental intent from beamline-specific implementation, the architecture enables consistent and reusable workflows across heterogeneous experimental environments.

### 4.4. Handling ambiguous and invalid requests

During beamline operation, user requests are often incomplete, ambiguous, or physically inconsistent. For example, users may omit key parameters, provide conflicting values, or request measurements that are not supported by the beamline. The orchestration framework is designed to handle these cases explicitly, preventing invalid actions and guiding the user toward valid experimental configurations.

The system operates under a constrained execution environment in which all actions must be validated before execution. As a result, no user request is executed directly. Instead, the AI agent first translates the request into structured capability calls and evaluates whether all required parameters are specified and physically consistent. When a request is incomplete, such as a scan command without a specified element or parameter range, the system does not attempt generating the plan. Instead, it requests clarification from the user, ensuring that all necessary inputs are explicitly defined.

For physically inconsistent inputs, the system leverages embedded domain knowledge to resolve the inconsistency where possible. For example, if a user requests an Mn XANES scan at an incorrect energy (*e.g.* 25 keV), the system identifies the mismatch and determines the appropriate Mn *K*-edge energy (approximately 6539 eV). A corrected scan range is then proposed and presented to the user for confirmation rather than executed directly.

In cases where a request falls outside the supported capability space, such as requiring functionality not available on a given beamline, the system does not generate an executable plan. Instead, it informs the user of the limitation and may propose alternative workflows that are compatible with the available configuration.

Combined with human-in-the-loop approval, this design ensures that all AI-assisted workflows remain transparent, physically meaningful, and consistent with established beamline safety practices. This approach enables flexible interaction while maintaining strict control over execution, ensuring that the system assists in experimental planning without compromising reliability or safety.

## 5. Conclusion

We have presented an AI-assisted orchestration framework for synchrotron beamline operation, demonstrated at the ALS beamline 5.3.1. By introducing a capability-based abstraction, the framework decouples experimental intent from beamline-specific implementation, enabling portable and consistent interaction across different instruments. The proposed architecture integrates AI-assisted workflow composition with graphical interfaces for monitoring and control, while maintaining safe operation through constrained execution within existing control systems. Together, these elements enable users to specify experiments at a high level, reducing the need for manual scripting while preserving visibility and control during execution. The demonstrated case studies show that this approach supports realistic experimental workflows, adapts to different beamline configurations, and avoids invalid or unsupported operations through context-aware planning.

Overall, this work establishes a practical and scalable pathway for integrating AI into synchrotron experimentation, positioning AI as a tool for orchestration and decision support rather than direct control. This approach improves accessibility and efficiency while remaining compatible with existing infrastructure and safety requirements.

## Supplementary Material

The evaluation methodology, measured LLM token usage, session-management strategies, and projected token consumption for a representative 60-sample beam time using the Osprey agentic system at ALS beamline 5.3.1. DOI: 10.1107/S1600577526007836/soo5005sup1.pdf

## Figures and Tables

**Table 1:** Representative capabilities in the AI-assisted orchestration framework

Capability	Key parameters	Description
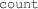	Detectors, number of readings	Acquire repeated detector measurements
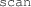	Detectors, motor, start, stop, steps	Perform motor scan with detector acquisition
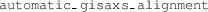	–	Execute automated GISAXS alignment
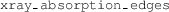	Chemical elements	Retrieve X-ray absorption edges from database
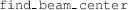	Image, mask (optional)	Find beam center from detector image
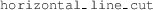	Image, center, width	Extract horizontal intensity profile

## Data Availability

The source code is available on GitHub: https://github.com/mlexchange/531_agents.

## References

[bb1] Allan, D., Caswell, T., Campbell, S. & Rakitin, M. (2019). *Synchrotron Radiat. News* **32**(3), 19–22.

[bb2] Beaucage, P. A. & Martin, T. B. (2023). *Chem. Mater.* **35**, 846–852.

[bb3] Bluesky Project (2026). *Tiled: secure, structured access to scientific data*, https://github.com/bluesky/tiled. Accessed: 2026–06–15.

[bb4] Chantler, C. T., Bunker, G., D’Angelo, P. & Diaz-Moreno, S. (2024). *Nat. Rev. Methods Primers* **4**, 90.

[bb5] Chen, Z., Petsch, A., Israelski, A., Plumley, R., Shen, L., Wang, C., Peng, C., Ni, Y., Bansil, A., Chowdhury, S., Li, M., Thayer, J., Thampy, V. & Turner, J. (2025). *An agentic artificially intelligent X-ray scientist*. Preprint, Research Square. Version 1,https://doi.org/10.21203/rs.3.rs-7456716/v1.

[bb6] Corrao, A. A., Maffettone, P. M., Ravel, B., Caswell, T. A., Campbell, S. I., Joress, H., Wilkins, S. & Olds, D. (2025). *arXiv*:2509.22959.

[bb7] EPICS Collaboration (2026). *EPICS – The Experimental Physics and Industrial Control System* Accessed: 2026–05–11.

[bb8] Guijarro, M., Felix, L., De Nolf, W., Meyer, J. & Götz, A. (2023). *Synchrotron Radiat. News* **36**(6), 12–19.

[bb9] Hellert, T., Abramov, D., Ajami, M., Barnard, E. S., Bhimji, W., Bhardwaj, G., Chavez, T., Daoud, H., Dunn, T. J., Fagnan, K., Farrell, S., Machado Gazolla, J. G. F., Luka, G., Damian, G., Jinghua, G., Ha, Y., Hexemer, A., Hoschouer, H., Xiangyang, J., Kadidia, K., Martino, G., Dylan, M., Miller, M. O., Miskovich, S. A., Myint, P., Naseem, S., Northen, T., Patrou, M., Pérez, G. E., Quinn, P., Rafique, H., Ratner, D., Reed, A., Reyes, E., Shang, H., Smith, M., Sulc, A., Tennant, C., Tripathi, P. K., Underwood, R., Wall, M., Wang, D., Wu, A., Xu, C., Yager, K. G. & Zhang, Z. (2026*a*). *Synchrotron Radiat. News* **39**(2), 42–48.

[bb10] Hellert, T., Montenegro, J. & Sulc, A. (2026*b*). *Mach. Learn.* **4**, 016103.

[bb11] Henke, B. L., Gullikson, E. M. & Davis, J. C. (1993). *At. Data Nucl. Data Tables* **54**, 181–342.

[bb12] Kaiser, J., Lauscher, A. & Eichler, A. (2025). *Sci. Adv.* **11**, eadr4173. 10.1126/sciadv.adr4173 PMC11691695 39742494

[bb13] Koepp, W., Sochor, B., McReynolds, D., Chavez, T., Noack, M., Sriramoju, R. V., Coffey, A. H., Wang, Y., Henn, E., Sambale, A. K., Euchler, E., English, D., Schlünzen, F., Schaible, E., Zhu, C., Koyiloth Vayalil, S., Roth, S. V. & Hexemer, A. (2026). *Photon Sci.* **1**, 330–342.

[bb14] Lawrence Berkeley National Laboratory (2026). *CBorg – Multi-Model AI Portal for Berkeley Lab*, https://cborg.lbl.gov/. Accessed July 2026.

[bb15] Leon, S. D., Kaufman, A., Hexemer, A., Islegen-Wojdyla, A., McReynolds, D., Crumlin, E., Iliev, K., Manha, N., Glans, P., Hamlyn, R., Allan, D., Gann, E., Rodolakis, F. & Mahl, J. (2025). *Proceedings of the 20th International Conference on Accelerator and Large Experimental Physics Control Systems (ICALEPCS­2025)*, Chicago, IL, USA, pp. 1795–1800. THPD088.

[bb16] Mathur, S., der Vleuten, N., Yager, K. G. & Tsai, E. H. R. (2025). *Mach. Learn.: Sci. Technol.* **6**, 025051.

[bb17] Morris, T. W., Rakitin, M., Du, Y., Fedurin, M., Giles, A. C., Leshchev, D., Li, W. H., Romasky, B., Stavitski, E., Walter, A. L., Moeller, P., Nash, B. & Islegen-Wojdyla, A. (2024). *J. Synchrotron Rad.* **31**, 1446–1456. 10.1107/S1600577524008993 PMC11542659 39466695

[bb18] Newville, M., easyXAFS Whittington, N., Levantino, M., Schlepuetz, C., Aleksei Guenzing, D., Rakitin, M., Kim, S.-W., kalvdans & De Nolf, W. (2025). *xraypy/xraydb: 4.5.8*, https://doi.org/10.5281/zenodo.16114067.

[bb19] NSLS-II (2025*a*). *Bluesky queue server API: Python client for the queue server*, https://github.com/bluesky/bluesky-queueserver-api. Accessed: 2026–04.

[bb20] NSLS-II (2025*b*). *Finch: a react component library for bluesky beamlines*, https://github.com/bluesky/finch. Accessed: 2026–04.

[bb21] Oberthür, D., Hakanpää, J., Chatziefthymiou, S., Pompidor, G., Bean, R., Chapman, H. N. & Weckert, E. (2025). *J. Synchrotron Rad.* **32**, 474–485. 10.1107/S1600577525000669 PMC11892905 39964790

[bb22] Prince, M. H., Chan, H., Vriza, A., Zhou, T., Sastry, V. K., Luo, Y., Dearing, M. T., Harder, R. J., Vasudevan, R. K. & Cherukara, M. J. (2024). *NPJ Comput. Mater.* **10**, 251.

[bb23] Voronov, D. L., Park, S., Islegen-Wojdyla, A., Salmassi, F. & Padmore, H. A. (2025). *Opt. Lett.* **50**, 3565–3567. 10.1364/OL.561082 40445653

[bb24] Vriza, A., Prince, M. H., Zhou, T., Chan, H. & Cherukara, M. J. (2026). *npj Comput. Mater.* **12**, 160.

[bb25] Wang, C., Kim, Y.-J., Vriza, A., Batra, R., Baskaran, A., Shan, N., Li, N., Darancet, P., Ward, L., Liu, Y., Chan, M. K. Y., Sankaranarayanan, S. K. R. S., Fry, H. C., Miller, C. S., Chan, H. & Xu, J. (2025). *Nat. Commun.* **16**, 1498. 10.1038/s41467-024-55655-3 PMC11833048 39962040

[bb26] Zhang, H., Pincelli, T., Jozwiak, C., Kondo, T., Ernstorfer, R., Sato, T. & Zhou, S. (2022). *Nat. Rev. Methods Primers* **2**, 54.

