# Supplementary material for: Orchestrating agentic systems at ALS beamline 5.3.1 with Osprey and Bluesky
Source: J Synchrotron Radiat. 2026 Sep 1;33(Pt 5):1382–91. doi: 10.1107/S1600577526007836 (PMC13549354; doi:10.1107/S1600577526007836)
Supplement: Supplementary file 1 [file s-33-01382-sup1.pdf]

JOURNAL OF SYNCHROTRON RADIATION

Volume 33 (2026)

Supporting information for article:

Orchestrating agentic systems at ALS beamline 5.3.1 with *Osprey* and *Bluesky*

Ka Hung Chan, Yang Ha, Antoine Islegen-Wojdyla, Seij De Leon, Manuel Garces, Dylan McReynolds, Xiaoya Chong, Raja Vyshnavi Sriramoju, Johannes Mahl, Damian Guenzing, Wiebke Koepp, Alexander Hexemer, Thorsten Hellert and Tanny Chavez

Orchestrating Agentic Systems at ALS Beamline 5.3.1 with Osprey and Bluesky

Ka Hung Chan, Yang Ha, Antoine Islegen-Wojdyla, Seij De Leon, Manuel Garces, Dylan McReynolds, Xiaoya Chong, Raja Vyshnavi Sriramoju, Johannes Mahl, Damian Guenzing, Wiebke Koepp, Alexander Hexemer, Thorsten Hellert, and Tanny Chavez

(Dated: Jul 2026)

I. EVALUATION OF LLM TOKEN USAGE

TABLE I. Measured token usage and projected consumption for a representative 60-sample beamtime.

Session strategy	Input tokens/cycle	Input tokens/60-sample	Output tokens/60-sample
Reset every sample	$75{,}645 \pm 2{,}365$	~ 4.5 million	~ 0.21 million
Reset every five samples	$89{,}944 \pm 16{,}051$	~ 5.1–5.4 million	~ 0.23–0.25 million
Continuous session	$98{,}938 \pm 5{,}122$	~ 5.9 million	~ 0.27 million

A. Evaluation procedure

LLM token usage was evaluated using a representative beamline workflow consisting of sample alignment, XANES scan generation and approval, and data retrieval. Large-language-model inference was provided remotely through the CBorg AI platform [1]. The reported token counts include all internal LLM calls made by the evaluated multi-model configuration, in which Anthropic Claude Sonnet 4.6 was used for workflow orchestration and OpenAI GPT-4o was used for the remaining agent components.

Two tests were performed. First, we evaluated run-to-run repeatability across five independent sessions, with each session including one follow-up interaction. Second, the three-step workflow was repeated for nine cycles using three different session-management strategies: (i) restarting the session for every sample, (ii) restarting the session after every five samples, and (iii) maintaining one continuous session. Input tokens, output tokens, and internal LLM calls were recorded for all agent components.

B. Measured token usage

Across the five independent sessions, the workflow consumed 97,050 ± 10,585 input tokens and 4,421 ± 470 output tokens per session. The token usage variation were approximately 11% for both input and output tokens, indicating that the overall token usage was reasonably consistent across repeated executions. When averaged across individual user interactions, the average usage was approximately 24,000 input tokens and 1,100 output tokens per interaction.

Session management primarily affected the steady-state input-token usage. Restarting the session for each sample consumed 75,645 ± 2,365 input tokens per sample measurement cycle. Restarting every five samples resulted in an intermediate but more variable usage of 89,944 ± 16,051 input tokens per cycle, whereas maintaining one continuous session consumed 98,938 ± 5,122 input tokens per cycle. No sustained increase in token usage was observed over the nine tested cycles. Instead, the continuous session reached an approximately stable level after the initial cycle.

C. Projected beamtime usage

Based on the measured steady-state values, a representative 60-sample beamtime is projected to consume approximately 4.5 million input tokens and 0.21 million output tokens when the session is restarted for each sample. Restarting the session every five samples gives an intermediate estimate of approximately 5.1–5.4 million input tokens and 0.23–0.25 million output tokens. Maintaining one continuous session increases the projected usage to approximately 5.9 million input tokens and 0.27 million output tokens.

Thus, restarting the session between independent samples reduces input token usage by approximately 25% relative to maintaining one continuous session. Continuous session memory may nevertheless be useful when decisions for later samples depend on context accumulated from previous measurements. Consequently, selecting a session strategy requires balancing token efficiency against the system's ability to retain context from previous measurement.

---

[1] Lawrence Berkeley National Laboratory, IT Division, CBorg AI Portal, https://cborg.lbl.gov/ (2026), accessed July 2026.
